# Chemoproteomic Covalent Ligand Discovery as the PROTAC-gonist: The Future of Targeted Degradation Medicines

**DOI:** 10.1021/acscentsci.4c01093

**Published:** 2024-07-16

**Authors:** Kyosuke Shishikura, Megan L. Matthews

**Affiliations:** Department of Chemistry, University of Pennsylvania, Philadelphia, Pennsylvania 19104-6243, United States

Targeted protein degradation (TPD) enables the levels of disease-relevant proteins to be therapeutically manipulated with small-molecule ligands. TPD can target proteins that cannot usefully be engaged by conventional small-molecule inhibitors. Many of these “undruggable” targets have important roles in cancer and the progression of other diseases. TPD is thus a promising new therapeutic modality, but challenges associated with its implementation to date have threatened to limit its generality. In this issue of *ACS Central Science*, Daniel Nomura and his team introduce a new approach to degrader design that promises to overcome these challenges and accelerate the discovery of TPDs and their implementation as drugs.^1^

**Figure 1:**
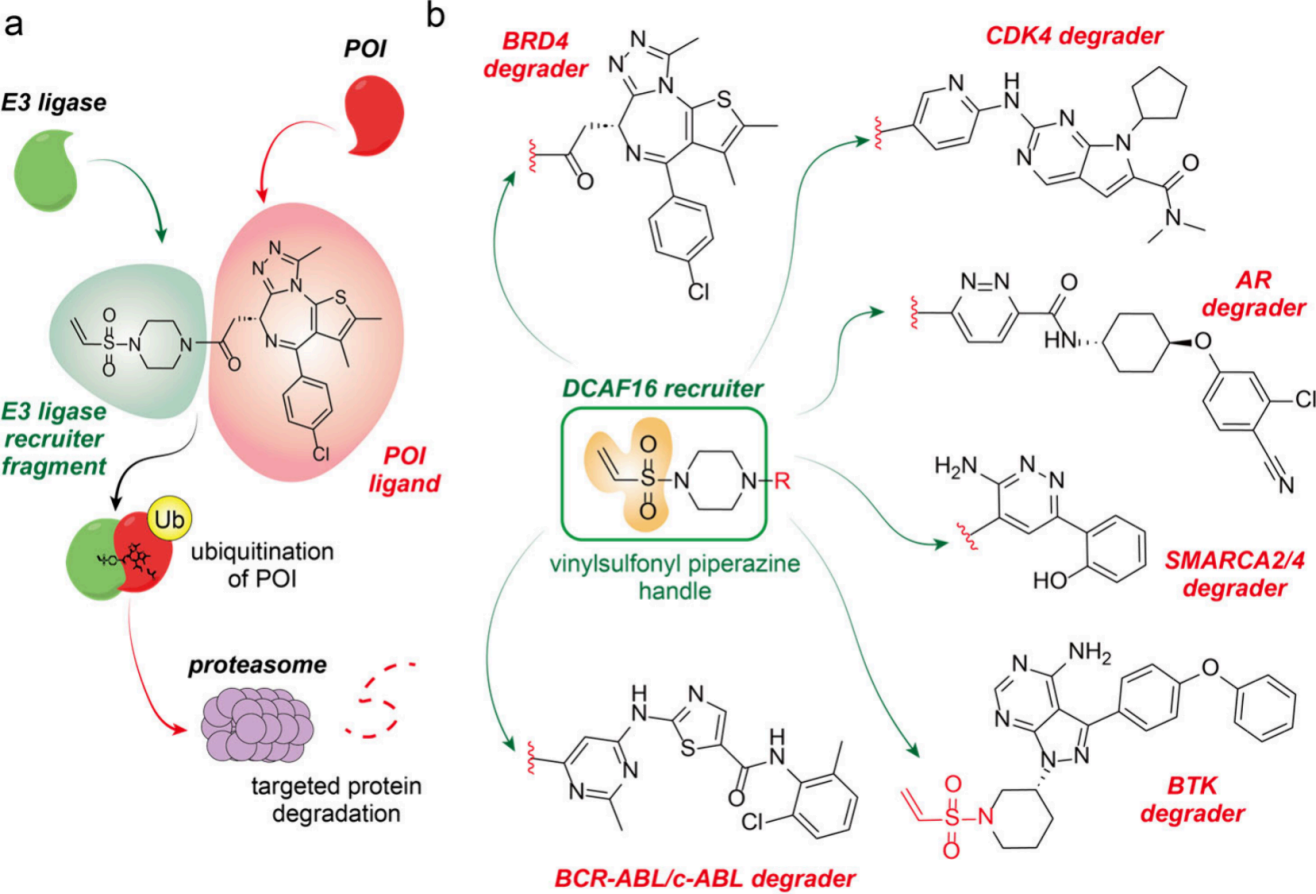
(a) Mechanistic scheme of molecular glue degrader. (b) The authors developed a transplantable handle that recruits DCAF16, an E3 Ub ligase. Six selective degraders were developed in the study using the general DCAF16 covalent recruiter handle.

TPD is particularly effective for intractable targets such as those with unusually broad, shallow, or featureless active sites or those where small molecule binding only modestly impacts activity. TPD inducers are of two main types: bivalent degraders (BVD), such as proteolysis targeting chimeras (PROTACs), and monovalent degraders (MVD), such as molecular glue degraders. PROTACs and molecular glue degraders work by the same mechanism, having two features in common; one ligand binds to the protein to be degraded (protein of interest, POI), while the other ligand (or ligand fragment) binds and recruits an E3 ubiquitin (Ub) ligase.^3^ The approximation of the POI and E3 promotes ubiquitination of the POI, initiating its proteasomal degradation. The main differences between PROTACs and molecular glue degraders are the flexibility in the design and size of the molecules. The modularity of PROTACs makes them highly amenable to rationale design: a tight binder for any given POI can be joined with one of several known E3 recruiters by a linker of appropriate length.^4^ Here, the efficacy of the molecule depends on the recruiter, the POI-ligand, and the linker, mandating that all three elements be optimized independently to effectively engage the ligase and POI in a configuration that successfully promotes Ub transfer to the POI. Due to this modular design, PROTAC degraders tend to be, and necessarily are, much larger than typical small molecule drugs, which poses challenges for oral delivery, central nervous system exposure, formulation, and metabolite-related toxicities.^4^ Because late-stage challenges with bioavailability originate from limitations intrinsic to the design strategy of PRTOACs, the true potential of targeted degradation therapies have remained limited in scope. By contrast, molecular glue degraders^4^ are single linker-less small molecules, recruiting and bridging an E3 ubiquitin ligase to a POI yet here with even closer proximity, thereby more efficiently inducing ubiquitination and degradation of the POI (Figure 1a).^5^ These characteristics make them comparable in size to typical drugs (Table 1), allowing for efficient delivery similar to that of conventional small molecule therapeutics. Molecular glue degraders are also advantageous for targeting less tractable proteins with shallow binding pockets, where PROTACs often fall short. Despite their promising therapeutic potential, de novo discovery of glue degraders has been either serendipitous or relied on high-throughput screening due to the lack of rationale design strategies. As such, further development and validation have relied on the requirement for interdisciplinary studies involving X-ray crystallography, molecular docking, and structure–activity relationship studies.^5^

**Table 1:**
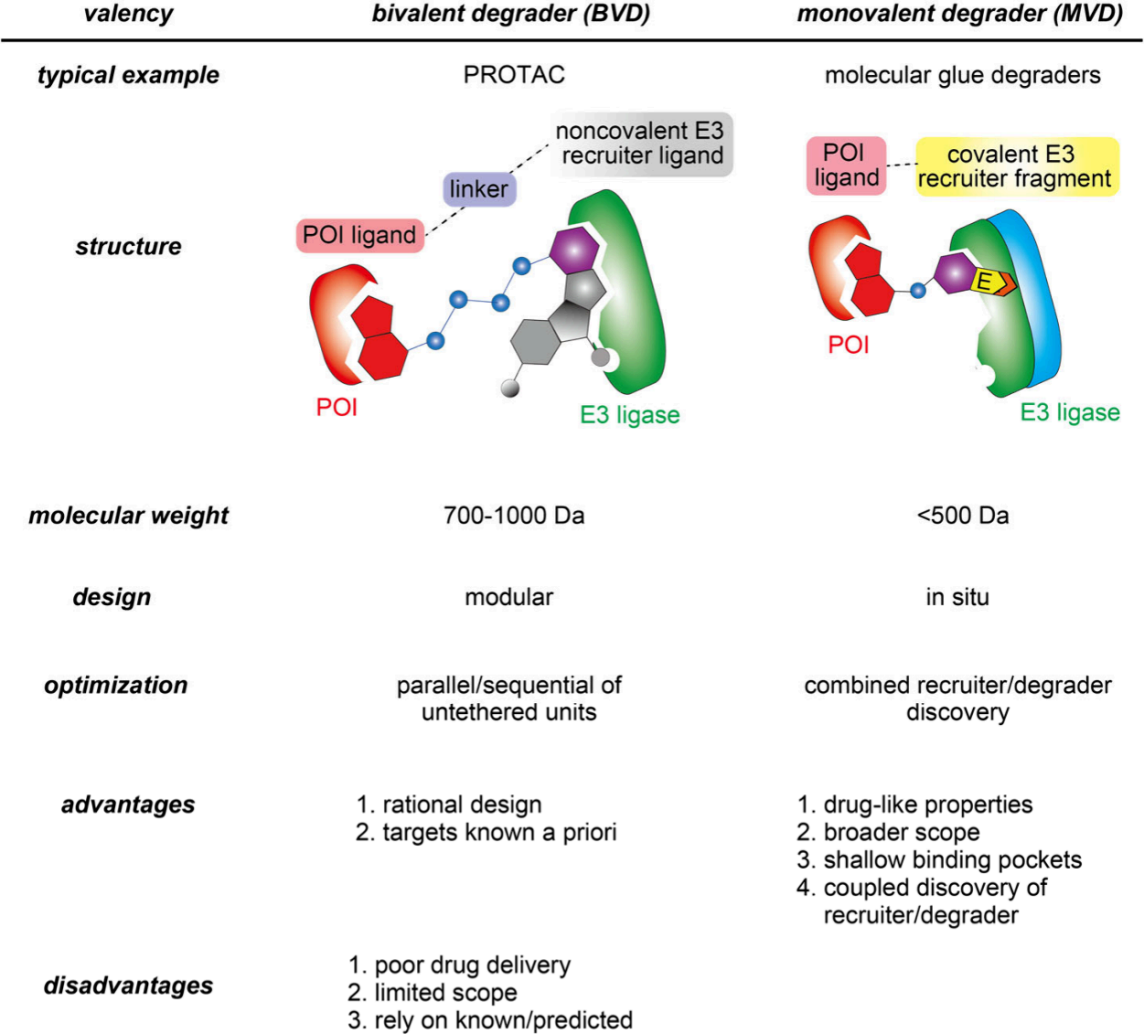
Comparison of Targeted Protein Degraders

Hints for the rational discovery of molecular glue degraders have slowly emerged. Abundant knowledge of E3 recruiters and their fragments have accumulated through structure-based molecule design and electrophile fragment screening by activity-based protein profiling (ABPP).^6^ ABPP serves as a mature and robust approach to discover covalent drugs/ligands by identifying the targets of covalent fragment libraries using chemical probes coupled to mass spectrometry-based proteomics (“chemoproteomics”). Continuing through the development of PROTACs, this area of chemical biology has indeed mastered the ability to develop selective, high-affinity ligands for nearly any protein, in other words “drugging the undruggable”, some for which may evoke a therapeutic effect. Lastly, several recent examples of successful transformations of nondegrative small molecule ligands into molecular glue degraders have been achieved by subtle chemical alterations that effectively recruit E3 ubiquitin ligases.^7,8^ To begin, the Nomura group appended 18 covalent fragments (putative E3 ligands) onto JQ1, a selective inhibitor of the POI, BRD4. Among the 18 JQ1 covalent handles, only the vinylsulfonyl piperazine (VSP) group modified the function of JQ1 from a binder to a binder-degrader of BRD4. Appending the VSP handle with a clickable alkyne group for proteome-wide target identification in cells via ABPP approaches, the authors discovered that DCAF16 was the primary ubiquitin E3 ligase recruited by the VSP electrophilic fragment that induced targeted degradation of BRD4 (Figure 1a). Successively, the VSP handle could also be appended to at least five other cancer-related nondegrative small molecule ligands to degrade their corresponding target proteins, each from unique structural and functional classes: 1. Cyclin-dependent kinase 4 (CDK4), 2. SWI/SNF related, matrix-associated, actin-dependent regulator of chromatin, subfamily a, member 2/4 (SMARCA2/4), 3. The androgen receptor (AR), 4. Breakpoint cluster region-abelson murine leukemia (BCR-ABL)/c-ABL, and 5. Bruton tyrosine kinase (BTK). These data demonstrate that the VSP handle is transplantable and can be used to design a general molecular glue degrader with a broad substrate scope via the covalent recruitment of the E3 ubiquitin ligase DCAF16 (Figure 1b).

The major technological advance made here by the Nomura group is that E3 ligase recruiters no longer need to be known a priori; they can now be discovered de novo and in tandem while already tethered directly (not by a linker) to a ligand that binds a POI. The Nomura group showed that by appending a small library of covalent electrophilic fragments to a given POI-ligand via a linker-less or near linker-less appendage, the foundational principles of ABPP and covalent fragment-based ligand discovery can be applied to the TPD drug modality. This approach allows for the discovery of the covalent E3 ligase recruiter and exploits the benefits of covalent mechanism drugs (via the E3 recruiter), which ABPP has repeatedly shown can successfully furnish potent, selective covalent inhibitors of enzyme active sites and ligandable binding pockets beyond traditional active site architectures.

Transforming nondegrative small molecule ligands into glue degraders is not completely new.^7−10^ However, for the vast majority of ligandable pockets and specific protein-small molecule interactions that are discovered, there is no general roadmap for conducting global counter screens that select for or against ligand interactions that perturb a protein’s primary function and/or that evoke a therapeutic effect. TPD ensures that there will be at least one, and the Nomura group showcases here that the scope and generalizability have the capacity to bind and degrade any ligandable POI across the proteome. The authors noted that off-targets of these glue degraders included the oncogene and cancer-associated protein Kelch-like ECH-associated protein 1 (KEAP1), a specific component of a ligase activity that regulates the Nrf2 transcription factor and expression of the antioxidant response gene element. Further improvement of selectivity and potency of these converted covalent degraders is needed; however, ABPP applied in a competitive format for inhibitor or ligand discovery and optimization provides a robust and streamlined process for future development and improvement.

Overall, the Nomura group has unveiled a new technology founded on the design principles of ABPP and demonstrated its utility in solving the PROTAC problem—this PROTAC-gonist approach developed by the Nomura group—is a versatile strategy for the design of molecular glue degraders with drug-like properties that are broader in scope and that have greater utility by recruiting E3 ligases by covalent mechanisms.
